# Targeting Wnt signaling for improved glioma immunotherapy

**DOI:** 10.3389/fimmu.2024.1342625

**Published:** 2024-02-21

**Authors:** Margarita Gutova, Jonathan C. Hibbard, Eric Ma, Heini M. Natri, Vikram Adhikarla, Nyam-Osor Chimge, Runxiang Qiu, Cu Nguyen, Elizabeth Melendez, Brenda Aguilar, Renate Starr, Holly Yin, Russel C. Rockne, Masaya Ono, Nicholas E. Banovich, Yate-Ching Yuan, Christine E. Brown, Michael Kahn

**Affiliations:** ^1^ Department of Stem Cell Biology and Regenerative Medicine, City of Hope Beckman Research Institute, Duarte, CA, United States; ^2^ Department of Hematology & Hematopoietic Cell transplantation (T cell Therapeutic Research Laboratories), City of Hope Beckman Research Institute, Duarte, CA, United States; ^3^ Translational Genomics Research Institute (TGen), Phoenix, AZ, United States; ^4^ Division of Mathematical Oncology, Department of Computational and Quantitative Medicine, City of Hope Beckman Research Institute, Duarte, CA, United States; ^5^ Cancer Biology and Molecular Medicine, City of Hope Beckman Research Institute, Duarte, CA, United States; ^6^ National Cancer Center, Tokyo, Japan; ^7^ Department of Computational and Quantitative Medicine, City of Hope Beckman Research Institute, Duarte, CA, United States

**Keywords:** glioma, Wnt signaling, pathway, ICG-001, immunotherapy, NanoString gene expression, proteomics, differentiation

## Abstract

**Introduction:**

Despite aggressive standard-of-care therapy, including surgery, radiation, and chemotherapy, glioblastoma recurrence is almost inevitable and uniformly lethal. Activation of glioma-intrinsic Wnt/β-catenin signaling is associated with a poor prognosis and the proliferation of glioma stem-like cells, leading to malignant transformation and tumor progression. Impressive results in a subset of cancers have been obtained using immunotherapies including anti-CTLA4, anti-PD-1, and anti-PD-L1 or chimeric antigen receptor (CAR) T cell therapies. However, the heterogeneity of tumors, low mutational burden, single antigen targeting, and associated antigen escape contribute to non-responsiveness and potential tumor recurrence despite these therapeutic efforts. In the current study, we determined the effects of the small molecule, highly specific Wnt/CBP (CREB Binding Protein)/β-catenin antagonist ICG-001, on glioma tumor cells and the tumor microenvironment (TME)–including its effect on immune cell infiltration, blood vessel decompression, and metabolic changes.

**Methods:**

Using multiple glioma patient-derived xenografts cell lines and murine tumors (GL261, K-Luc), we demonstrated *in vitro* cytostatic effects and a switch from proliferation to differentiation after treatment with ICG-001.

**Results:**

In these glioma cell lines, we further demonstrated that ICG-001 downregulated the CBP/β-catenin target gene *Survivin/BIRC5–*a hallmark of Wnt/CBP/β-catenin inhibition. We found that in a syngeneic mouse model of glioma (K-luc), ICG-001 treatment enhanced tumor infiltration by CD3^+^ and CD8^+^ cells with increased expression of the vascular endothelial marker CD31 (PECAM-1). We also observed differential gene expression and induced immune cell infiltration in tumors pretreated with ICG-001 and then treated with CAR T cells as compared with single treatment groups or when ICG-001 treatment was administered after CAR T cell therapy.

**Discussion:**

We conclude that specific Wnt/CBP/β-catenin antagonism results in pleotropic changes in the glioma TME, including glioma stem cell differentiation, modulation of the stroma, and immune cell activation and recruitment, thereby suggesting a possible role for enhancing immunotherapy in glioma patients.

## 1 Introduction

Despite aggressive standard of care therapy, including surgery, radiation, and chemotherapy, glioblastoma (GBM) recurrence is almost inevitable and uniformly lethal (1–3). In glioma, Wnt pathway activation has been associated with a poor prognosis and progressive neurological deficits (4). Wnt signaling is associated with the proliferation of stem-like cells (5–7) as well as stark resistance to chemotherapy, radiotherapy, and immunotherapy in GBM (5–9). Unbiased profiling studies demonstrate a strong negative correlation between cancer cell stemness and antitumor immunity signatures across 21 types of solid tumors, with reduced anticancer immune cell tumor infiltration (i.e., CD8^+^ T cells, natural killer cells, and B cells) and increased tumor-associated macrophages (10). β-catenin transcriptional activation, involving its translocation to the nucleus, is a hallmark of Wnt pathway activation and has been identified in 19% of adult and in 30% of pediatric gliomas (11). A resistance mechanism observed in immunologically “cold tumors”, including gliomas, involves aberrant activation of the Wnt/β-catenin signaling pathway (12, 13). Enhanced tumor-intrinsic Wnt/β-catenin signaling appears to be a common mechanism mediating cancer immune evasion and is associated with the presence of an immunosuppressive cell subset and the prevention of effective dendritic cell presentation and T-effector cell recruitment and function (14). Increased expression of β-catenin inversely correlates with the presence of CD8^+^ T cells and dendritic cells in multiple tumor types, including glioma (13, 15). Furthermore, Wnt pathway activation is correlated with tumor stemness, hypoxia, and poor treatment outcome (16, 17). The hostile tumor microenvironment (TME) is associated with decreased tumor antigen presentation and reduced or lost efficacy of various therapies, including adoptive T cell immunotherapy (18–22). Therefore, targeted downregulation of Wnt/β-catenin signaling–thereby enhancing the response to immunotherapy in patients with relapsed and refractory tumors–is an attractive therapeutic approach.

Glioma stem cells (GSC), via secretion of the Wnt‐induced signaling protein 1 (WISP1), can further facilitate a ‘cold’ TME by promoting the survival of both GSC and tumor-associated macrophages (TAM) (14). Activation of Wnt/β-catenin signaling causes tumor cell proliferation, enhanced invasiveness via upregulation of JNK, and accumulation of metalloproteases, with concomitant neuronal degeneration due to decreased requisite Wnt signaling maintenance (23–25). Clinical and preclinical data suggest that curative immunotherapy must not only address immunotolerance and target tumor antigens, but also circumvent intrinsic and evolving barriers of adaptive and acquired immune escape mechanisms (12, 15). The hostile TME leads to a loss of therapeutic efficacy of immunotherapy, tumor antigen vaccination, and adoptive T cell transfer immunotherapy (including CAR T cell) approaches (13, 26–28). Support for the concept that increased Wnt/β-catenin activity plays a role in CAR T response in glioblastoma was provided by a patient who had a complete initial response to CAR T cell therapy; however upon relapse and antigen loss, tumor samples demonstrated the activation of several genes in the Wnt/β-catenin pathway (including Wnt 11 and Wnt 2A) (19, 29). Currently, WNT inhibitors in clinical trials include PORCN inhibitors, WNT ligand antagonists, FZD antagonists, and CBP/β catenin antagonists tested in various solid tumors and leukemia (30). The refractory nature of gliomas provides compelling motivation for the development of novel therapeutic interventions including CAR T cell therapy for glioma and other devastating malignancies (18, 19, 31–33). Taken together, our results demonstrate that inhibition of Wnt/CBP/β-catenin signaling can induce glioma cell differentiation *in vitro* and *in vivo*, modify the TME, and affect immune cell populations in the TME, shifting towards more effective dendritic cell presentation and a more effective T cell response, thereby potentially enhancing immunotherapeutic interventions in glioma patients.

## 2 Materials and methods

### 2.1 *In vitro* experiments

PBT tumor cells lines are derived from patients with brain tumors (IRB07074) dissociated and grown in DMEM/F12 medium, supplemented with heparin, hepes, glutamax, and B27. Epidermal growth factor (EFG) and fibroblast growth factor (FGF) are added at the time of culture, as described previously (34). ICG-001 was provided by M. Kahn’s laboratory. Co-culture assay of PBT cell lines were grown as described above and seeded to 100,000 cells/2 ml in 6-well plates. ICG-001 was added to cell cultures in concentrations 0, 5, and 10 µM for 24–72h, as described previously (35).

### 2.2 RT-PCR analysis

RNA was extracted using the Total RNA Kit according to the manufacturer’s instructions (Qiagen, RNeasy PowerSoil). cDNA was generated with the High-Capacity cDNA Reverse Transcription Kit (Applied Biosystems). Quantitative RT-PCR was performed using Power SYBR Green PCR Master Mix (Applied Biosystems). Amplification of human *Kif20A* was performed on RNA samples isolated from PBT147 and PBT030 cells treated with ICG-001 at concentrations of 0, 5, and 10 µM. All data was normalized to the PBT147-24H-0 drug control sample. Human *Kif20A* RT-PCR primers used for RT-PCR analysis were: Forward: TGGTACGCAAGAACCTGC; Reverse: GATCAGGGTTGTGTCCGT. Human GAPDH primers were used as controls: Forward: GGATTTGGTCGTATTGGG; Reverse: GGAAGATGGTGATGGGATT.

### 2.3 Proteomics data analysis

The normalized distributions of protein levels were heavily skewed to the right, so we performed a logarithmic transformation of the data, which resulted in an approximate normal distribution of protein levels. The densities of the log-transformed normalized protein level for each cell line/dose are shown in **Supplementary Figure 3**. To examine the effect of ICG-001, we calculated the difference in log normalized levels for each protein between the four treated samples and the appropriate untreated sample (PBT147 and PBT030 cell lines were treated with ICG001 at 0, 5, and 10 µM, and cells were collected after 24 or 72 h for protein analysis), which corresponds to examining the log of the fold changes in each protein after treatment. For each of the two cell lines (PBT147 and PBT030), we plotted the log fold change for each protein at 5µM against the log fold change at 10µM. These plots are shown in **Supplementary Figure 3A**, with the points colored-coded by cell line.

### 2.4 Animal studies

Anesthesia: For tumor models, mice were anesthetized by intraperitoneal (i.p.) injection of Ketamine/Xylazine and gaseous Isoflurane prior to tumor injection. Kluc [0.1×10^5^ were prepared in PBS−/− (2 μL per mouse)] and injected orthotopically in the brain parenchyma of female NSG mice via stereotactic injection. Tumor growth was monitored at least once a week via optical imaging (Spectral Instruments Imaging, LagoX) and flux signals were analyzed with Aura Imaging software (LagoX). For imaging, mice were injected intraperitoneally with 150 μL d-luciferin potassium salt (Perkin Elmer) suspended in PBS at 4.29 mg/mouse. Once flux signals reached desired levels, CAR T cells were prepared in PBS and mice were treated either by intratumoral/intracranial injection in 3 μL final volume. At desired time points or at moribund status, mice were euthanized, and tissues were processed for IHC as described below. Syngeneic mice (C57BL/6) of 8–12 weeks of age were implanted with subcutaneous K-Luc tumors (n=8). 7 days later, when tumors became palpable and after confirmation of tumor presence with BLU imaging, mice were implanted with Alzet minipumps. Pumps continuously provided a daily dose of ICG-001 (50 mg/kg/day). Tumor tissues were harvested on days 7, 14, and 21 post pump implantations, and tumors were prepared for IHC (paraffin sections) and NanoString analysis. Control mice were not treated with ICG-001 pumps, and tumors from control mice were harvested on day 7, 14 and 21 to match ICG-001-treated tumors.

### 2.5 Immunohistochemistry

**Figure 1:**
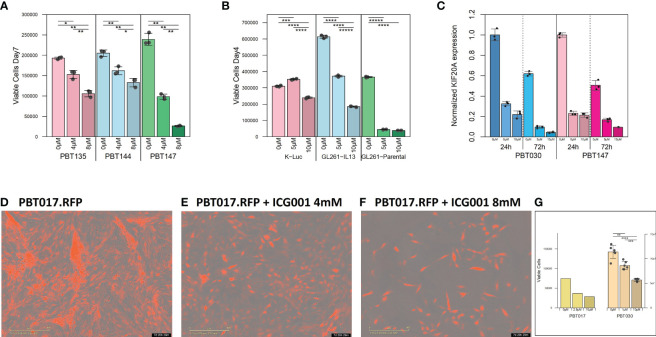
Cytostatic effect of ICG-001 in human and mouse glioma cell lines *in vitro*. **(A)** Growth kinetics of human PBT glioma lines (PBT135, PBT144, and PBT147) treated with ICG-001 (0, 5, 10 µM) for 7 days (n=3); and **(B)** mouse glioma cells (K-luc, GL261-IL13, and GL261) treated with ICG-001 (0-10 µM) for 4 days (n=3). Scale bars represent SD of triplicate samples from 2 independent experiments. **(C)** Expression of target gene KIF20 post-ICG-001 treatment (5, 10 µM) by RT-PCR using KIF20A-specific primers (n=3). **(D–F)** Live images taken by IncuCyte of PBT017 glioma expressing red fluorescent protein untreated or treated with ICG-001 (0, 4, 8 µM). **(G)** Analysis and quantification of IncuCyte images (10x) for PBT017.RFP (PBT017 expressing red fluorescent protein-RFP) glioma cells and PBT030 quantified using phase images. P values denoted by *<0.05, **<0.01, ***<0.001, ****<0.0001, *****<0.00001.

Three immune cell markers (CD3, CD8, CD31, Abcam) were evaluated with IHC on whole tumor sections. IHC Staining was performed in the City of Hope Pathology Core according to the manufacturer’s instructions (n=6 mice). After IHC staining, slides were scanned using the NanoZoomer 2.0-HT slide scanner at 10× magnification (Hamamatsu Photonics). Scanned slides were then imported into Qupath as Brightfield H-DAB images for analysis. Tumor section annotations were manually outlined with the brush and wand tools. Once all tumor areas were selected, total cell count, positive cell count per area, and total area were counted using the Positive Cell Detection tool with the settings optimized for each CD3, CD8 and CD31 staining (**Figure 1**). All data were extracted from QuPath and further calculations and quantifications were done with Microsoft Excel and Prism.

### 2.6 Qupath quantification method

After IHC staining, the slides were scanned using the NanoZoomer 2.0-HT slide scanner (Hamamatsu Photonics). Scanned slides were then imported into Qupath as *Brightfield H-DAB* images for analysis. Tumor section annotations were manually outlined with the brush and wand tool. Once all tumor areas were selected, total cell count, positive cell detection, positive %, and total area were counted using the Positive Cell Detection tool with the auto settings (**Figures 1D, H, L**). All data were then extracted from Qupath and further calculations and quantifications were done with Microsoft Excel and Prism.

### 2.7 NanoString data analysis

Raw gene count was averaged across three brain tissue samples from CAR T treated mice and across two brain tissue samples from CAR T + ICG001 treated groups. Brain tissue was isolated during euthanasia, and tumors were dissected and snap frozen in liquid nitrogen. Total RNA was isolated from K-Luc tumor tissue and analyzed by NanoString (NanoString nCount mouse PanCancer Immune profiling paneled assays, https://nanostring.com/products/ncounter-assays-panels/oncology/pancancer-immune-profiling). Multiplex gene expression analysis was performed in mice for 770 genes from different immune cell types, common checkpoint inhibitors, CT antigens, and genes covering both the adaptive and innate immune response. The panel measures many features of the immune response to facilitate rapid development of clinical actionable gene expression profiles in the context of cancer immunotherapy. Comparisons between brain tissue and ICG001-treated tissue were determined by the nonparametric *U*-test, and log_2_ |fold change| ≥ 1.5, -Log_10_ P-value < 0.05 using the Benjamini-Hochberg method to be statistically significant. Log2 normalized counts and expression ratios were generated using nSolver 4.0 and advanced analysis 2.0 (NanoString Technologies, Inc.) as well as ROSALIND analysis platform v3.38.0.1.

### 2.8 ROSALIND® NanoString gene expression methods

Data was analyzed by ROSALIND® version 3.38.0.1 (https://www.rosalind.bio/), with a HyperScale architecture developed by ROSALIND, Inc. (San Diego, CA). Read Distribution percentages, violin plots, identity heatmaps, and sample MDS plots were generated as part of the QC step. Normalization, fold changes and p-values were calculated using criteria provided by NanoString. ROSALIND® follows the nCounter® Advanced Analysis protocol of dividing counts within a lane by the geometric mean of the normalizer probes from the same lane. Housekeeping probes for normalization were selected based on the geNorm algorithm as implemented in the NormqPCR R library1. Abundance of various cell populations was calculated on ROSALIND using the NanoString Cell Type Profiling Module. ROSALIND was used to perform a filtering of Cell Type Profiling results to include results that have significant scores (p ≤ 0.05). Fold changes and p-values were calculated using the fast method, as described in the nCounter® Advanced Analysis 2.0 User Manual. P-value adjustment was performed using the Benjamini-Hochberg method of estimating false discovery rates (FDR). Clustering of genes for the final heatmap of differentially expressed genes was performed using the Partitioning Around Medoids (PAM) method using the fpc R library2 that takes into consideration the direction and type of all signals on a pathway (the position, role and type of every gene, etc.). Hypergeometric distribution was used to analyze the enrichment of pathways, gene ontology, domain structure, and other ontologies. The topGO R library3, was used to determine local similarities and dependencies between GO terms in order to perform Elim pruning correction. Several database sources were referenced for enrichment analysis, including Interpro4, NCBI5, MSigDB6,7, REACTOME8, and WikiPathways9. Enrichment was calculated relative to a set of background genes relevant for the experiment (ACTB, GAPDH).

## 3 Results

### 3.1 Cytostatic effect of ICG-001 in human and mouse glioma cell lines *in vitro*

We initially tested the effects of the specific small molecule CBP/β-catenin antagonist ICG-001 *in vitro* as a single agent on 5 human PBT and 3 mouse glioma lines. Treatment with ICG-001 (0–10 µM) showed a concentration-dependent cytostatic effect in all tested human patient-derived glioma cell lines (PBT017, PBT030, PBT135, PBT144, and PBT147) (**Figures 1A, D–F**) and all murine-derived glioma cell lines (K-luc, GL261-parental, and GL261.IL13Rα2 engineered) (**Figure 1B**). We found that the KIF20A gene was downregulated in the PBT030 and PBT147 lines upon treatment of glioma cells with ICG-001 (**Figure 1C**). KIF20A, a mitotic kinesin, plays an important role in controlling the mode of division of neural progenitor cells (NPC) in both normal brain cells and brain tumor cells, and KIF20A knockdown induces a transition from proliferative to differentiative divisions in NPC (36, 37). Consistent with ICG-001-induced differentiation of PBT147 and PBT030 lines, ICG-001 treatment led to a significant reduction in the expression of *KIF20A*, as measured by qPCR (**Figure 1C**) (36), further confirming that the differentiating effects of ICG-001 on glioma lines are not cell line dependent. Additionally, all glioma lines exhibited a more differentiated phenotype based on their elongated cell morphology, with decreased proliferation and loss of clonal expansion, as shown by the PBT017.RFP human glioma cell line, imaged and quantified by Incucyte (**Figures 1D–G**). We also correlated human Wnt gene family with CD3 mRNAseq expression using public TCGA GBM datasets and RNA-seq data [downloaded from the GDC portal (https://portal.gdc.cancer.gov/)] (**Supplementary Figures 1A, B**). Upregulation of the Wnt pathway in glioma was inversely correlated with CD3 cell infiltration (**Supplementary Figure 1B**). A strong negative correlation was found among Wnt genes-APC, AXIN1, AXIN2, GSK3BTCF4 and CD3 score and a positive correlation was found for LEF1, CDC42 and CD3.

### 3.2 ICG-001 specifically targets Wnt/CBP/β-catenin transcription in glioma

**Figure 2:**
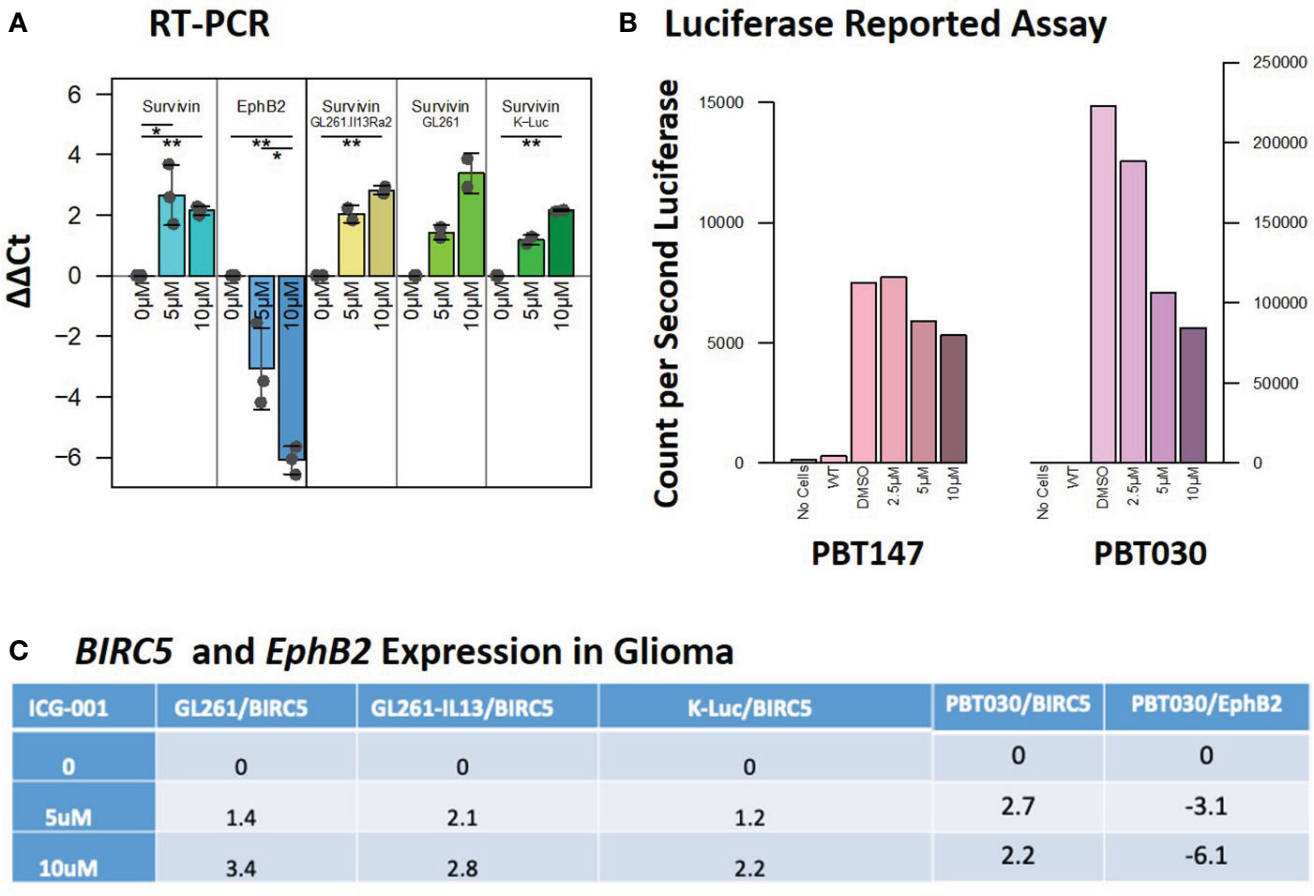
Expression of the target genes *BIRC5/Survivin* and *EphB2* post-ICG-001 treatment (0, 5, 10 mM) as detected by RT-PCR and Luciferase Reporter Assay. **(A)** Survivin and *EphB2* mRNA levels in PBT017 and PBT030 human glioma lines and mouse GL-261 and K-Luc lines treated with ICG-001 for 3 days (0, 5, 10 μM). **(B)** Luciferase reporter assay indicating downregulation of luciferase reported gene in PBT017 and PBT030 cell lines upon treatment with ICG-001 at 0, 5, and 10 μM in triplicates. **(C)** *Survivin/BIRC5* relative expression upon treatment with ICG001. P values denoted by *<0.05, **<0.01, ***<0.001, ****<0.0001, *****<0.00001.

Decreased expression of the Wnt/CBP/β-catenin target gene *BIRC5/Survivin*, with concomitant upregulation of the Wnt/p300/β-catenin target gene *EphB2*, is a hallmark of specific CBP/β-catenin inhibition (**Figure 2A**) (38). In our studies, we used *EphB2* and *BIRC5/Survivin* as a biomarker of the response to ICG-001. *BIRC5*/*Survivin* expression was also previously used as a biomarker for pharmacokinetic/pharmacodynamic (PK/PD) activity of the Wnt pathway in circulating tumor cells in the first-in-human clinical trial of the second-generation CBP/β-catenin specific antagonist PRI-724 (39). We demonstrated that *BIRC5/Survivin* gene expression was selectively downregulated and *EphB2* was upregulated in our experiments in both human and mouse glioma cell lines post ICG-001 treatment, as shown by qPCR, which was further confirmed using a Survivin-luciferase reporter assay for PBT017 and PBT030 cell lines *in vitro* (**Figure 2B**). ICG-001’s selective effects on the expression of *BIRC5/Survivin* and *EphB2* serve as an indicator of on target activity *in vitro* in the human and mouse G261, GL261.IL13Ra2, and K-luc cell lines (**Figures 2A, C**).

### 3.3 ICG-001 specifically targets glioma cell metabolism via Wnt/CBP/β-catenin modulation

**Figure 3:**
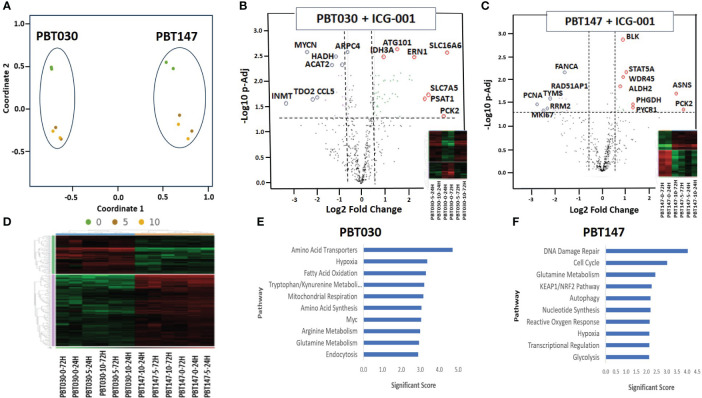
NanoString analysis of human metabolic pathways after treatment with ICG-001. **(A)** Multidimensional scaling (MDS) plot of visual representation of the patterns of proximities among PBT030 and PBT147 human cell lines treated with ICG-001 at 2 time points (24 hrs & 72 hrs) and 3 doses (0µM, 5µM, 10 µM) [n=2] separately on the Y-axis. X-axis indicated clustering of PBT030 and PBT147 cell lines each separately. **(B)** Volcano plot showing difference in expression for PBT030 between ICG-001 treated vs untreated, with the x-axis showing log2 fold change and the y-axis showing the negative log10 of p-adj value and heatmap; mRNA represented by red circles for significantly upregulated genes and blue circles for significantly down-regulated human genes. Full list of differentially expressed genes is shown in **Supplementary Table 2** **(C)** Volcano plot showing difference in expression for PBT147 between ICG-001 treated vs untreated, with the x-axis showing log2 fold change and the y-axis showing the negative log10 of p-adj value and heatmap; mRNA represented by red circles for significantly upregulated genes and blue circles for significantly down-regulated genes. Full list of differentially expressed genes are shown in **Supplementary Table 2**. **(D)** Heat map of differentially expressed genes when comparing PBT030 and PBT147 cell lines (selected glioma gene list). **(E, F)** Bar chart of functional analysis showing the top 10 significance score predicted using ROSALIND Gene Set Analysis with NanoString annotations for PBT030 and PBT147 human cell lines treated with ICG-001.

The N-termini of the two human Kat3 coactivators, CBP and p300, provide a highly evolutionarily conserved hub to integrate multiple signaling cascades that coordinate cellular metabolism with the regulation of symmetric versus asymmetric division of somatic stem cells and cancer stem cells, cellular proliferation, and differentiation status and function (40). More specifically, small molecule inhibition of the N-terminal region of CBP enhances p300/β-catenin mediated transcription, which is a prerequisite for increased mitochondrial oxidative metabolism during the initiation of cellular differentiation (40, 41). To explore ICG-001’s impact on metabolic changes associated with glioma differentiation after disrupting the CBP/β-Catenin interaction, we treated patient-derived GBM cell lines PBT147 and PBT030 with ICG-001 (0, 5, or 10 µM*)* for 24 and 72 h (**Figure 3**). RNA isolated from the treated PBT cells was analyzed using the NanoString nCounter metabolic panel (NanoString Technologies) (42). We used Rosalind software v.3.38.0.1 to analyze differential gene expression between PBT030 and PBT147 cell lines (**Figures 3A–D**). Furthermore, we analyzed gene and pathway expression in ICG-001 treated PBT samples as compared with untreated controls (**Figures 3E, F**). Statistically significant genes and pathways were analyzed using log_2_ Fold Change ≥ 1.5, < -1.5 and -Log_10_ p-Adj <0.05 filter. First, we demonstrated differences between PBT030 and PBT147 patient-derived lines (X-axis shift on MDS plot) and that ICG-001 treatment caused a similar shift in gene expression on the Y-axis among untreated and ICG-001-treated cell lines (**Figure 3A**). We detected genes upregulated in PBT030 cells, such as SOX2, MYCN, NOS2, RUNX2, and VEGFA, while PBT147 cells upregulated level of PTEN, STAT6, CA9 and TLR2, demonstrating differences among PBT cell lines, which can be explained by various driving mutations and heterogeneity in glioma lines (**Figures 3A–D** and **Supplementary Figures 2A, B**).

**Figure 4:**
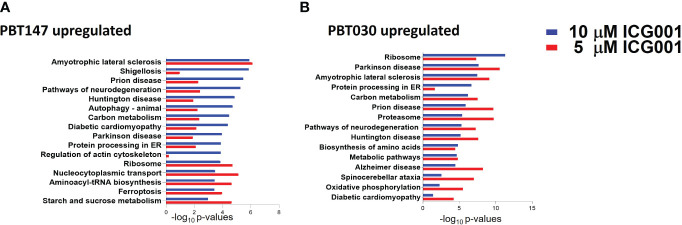
Proteomics analysis of PBT147 and PBT030 cell lines treated with ICG-001 (0-10 µM) for 72h. **(A)** Up-regulated protein density was calculated by the difference in log normalized levels for each protein between the four treated samples (ICG001 5, 10 mM at 24 and 72h) and the appropriate untreated sample control. **(B)** Calculating the difference in log levels corresponds to the log of the fold changes in each pathway. For both cell lines, the log fold-change is plotted for each protein at 5 µM against the log fold-change at 10 µM. These plots are shown in **(A)**, with the points color-coded by ICG001 concentrations. This highlights a positive linear relationship between the log fold changes at 5µM and 10µM in both cell lines. The dose effect is significantly greater in PBT030 than PBT147, with the same increase in dose, usually resulting in a greater fold-change.

Next, we tested if ICG-001 drives a similar metabolic shift in both lines. Fully consistent with our previous studies (40), we found similar changes in several genes and metabolic pathways after treatment of PBT030 and PBT147 with ICG-001–an increase in amino acid transporters, hypoxia, glutamine metabolism, MAPK, autophagy, mitochondrial respiration, cytokine and chemokine signaling, and arginine metabolism in both lines (**Supplementary Figure 7**). The PBT030 line demonstrated a decrease of tryptophan/kynurenine, fatty acid synthesis and oxidation, glycolysis, and the pentose phosphate pathway (**Supplementary Figure 7**). Similarly, in PBT147 lines treated with ICG-001, an increase in glutamine metabolism, autophagy, amino acid transport and synthesis, and MAPK, and strong down regulation in cell cycle, pentose phosphate, fatty acid synthesis, and oxidation was observed. (**Supplementary Figure 7**). This change in tumor cell metabolism may provide CD8 effector cells with the ability to effectively compete metabolically within the TME (43), which increases the likelihood of immune cell infiltration into the otherwise hostile TME (44). Consistent with our *in vitro* data (**Figures 1** and **2**), we also observed downregulation of cell cycle-related genes post ICG-001 treatment in PBT030 (PRIM2, NPM1) and PBT147 cell lines (including KIAA0101-PCNA, MKI67, RRM2, TYMS, CLSPN, CDCA8, UBE2C, EXO1, BRCA1, BRCA2, BRIP1, and PRIM1), which is associated with cytostasis and metabolic changes required for the switch from proliferation to differentiation (**Figures 3B, C**) (40). Fatty acid oxidation–the metabolic pathway preferred by quiescent stem-like cells (45)—was decreased in all treated cell lines at all timepoints and all doses. Furthermore, ALDH2 (PBT147), ACAT2, and HADH were downregulated in PBT030 cells, indicating that CBP/β-Catenin inhibition directs GSC activation and differentiation via metabolic reprogramming. These metabolic changes have been shown to be critical for the transition of quiescent to activated CSC (46) and their subsequent differentiation to bulk tumor cells (36). Upregulation of the p53 tumor suppressive pathway and mitochondrial respiration (up-PBT030-PPARGC1A, MCP1, PDP1, ME2, IDH3A, UQCR11, FAHD1, SOD2, and ATP6V1F), and glutamine metabolism (up-PBT030-PSAT1, GOT1, SERINC1; PBT147-ASNS, PYCR1, and PHGDH)—hallmarks of cell differentiation–were also observed at both time points, at both the 5- and 10-µM doses and in both cell lines (**Supplementary Figure 2**). A full list of up and downregulated genes with log_2_ Fold Change ≥ 1.5, < -1.5 and -Log_10_ p-Adj 0.05 filter is shown in **Supplementary Figure 2**. Selected data is shown for PBT147 and PBT030 in **Figures 4B, C, E, F**, and **Supplementary Figure 7**.

### 3.4 Proteomic analysis of glioma cell lines PBT147 and PBT030 upon treatment with ICG-001

To see the effect of ICG-001 treatment on the protein level in glioma cell lines, PBT147 and PBT030 cells were treated with ICG-001 at 0, 5, and 10 µM for 72 h (**Figure 4**). We collected cells and conducted global proteomic analyses after treatment (**Figure 4**). Using 2-DICAL, we detected 1,553 common proteins expressed in both PBT030 and PBT147 lines (47). The resulting data was quantile normalized and differential protein expression was further analyzed by ANOVA. Differentially expressed proteins were subjected to pathway enrichment analysis utilizing the KEGG database. Pathways with statistically significant enrichment (p-value <0.0001) following 5 µM or 10 µM ICG-001 treatments are represented in **Figure 4**. Both PBT147 and PBT030 cell lines demonstrated upregulation of pathways involved in neurogenesis, autophagy, regulation of the actin skeleton, and protein synthesis in the ER (**Figures 4A, B**). Consistent with the activation of quiescent stem-like cells, ICG-001 treatment of PBT147 and PBT030 induced metabolic reprogramming with increased ATP production, and an increase in protein translation and differentiation (48) (**Supplementary Figure 3**). Specifically, we found increased protein expression of APOD (lipid metabolism and neuroprotection), AAAS (metabolic protein transport), MFGM (promotes phagocytosis of apoptotic cells, wound healing), PDPR (metabolism), PGM1 (metabolism-glycosylation), RRAGA (metabolism), RRAGB (regulator of TOR signaling), RTC1(metabolism), SERC, and SYMC, and downregulation of MCM6 (cell cycle, DNA replication), GSTM5 (oxidative stress, metabolism), SORCN (Proto-oncogene), FADS2 (fatty acid metabolism), and PSD7 (cytoskeletal remodeling) upon treatment of PBT147 and PBT030 with ICG-001. We postulate that these metabolic changes in tumor cells post ICG-001 treatment, leading towards a more differentiated glioma phenotype, modulate the TME and influence CAR T cell efficacy.

### 3.5 ICG-001 specifically enhances tumor T cell recruitment

**Figure 5:**
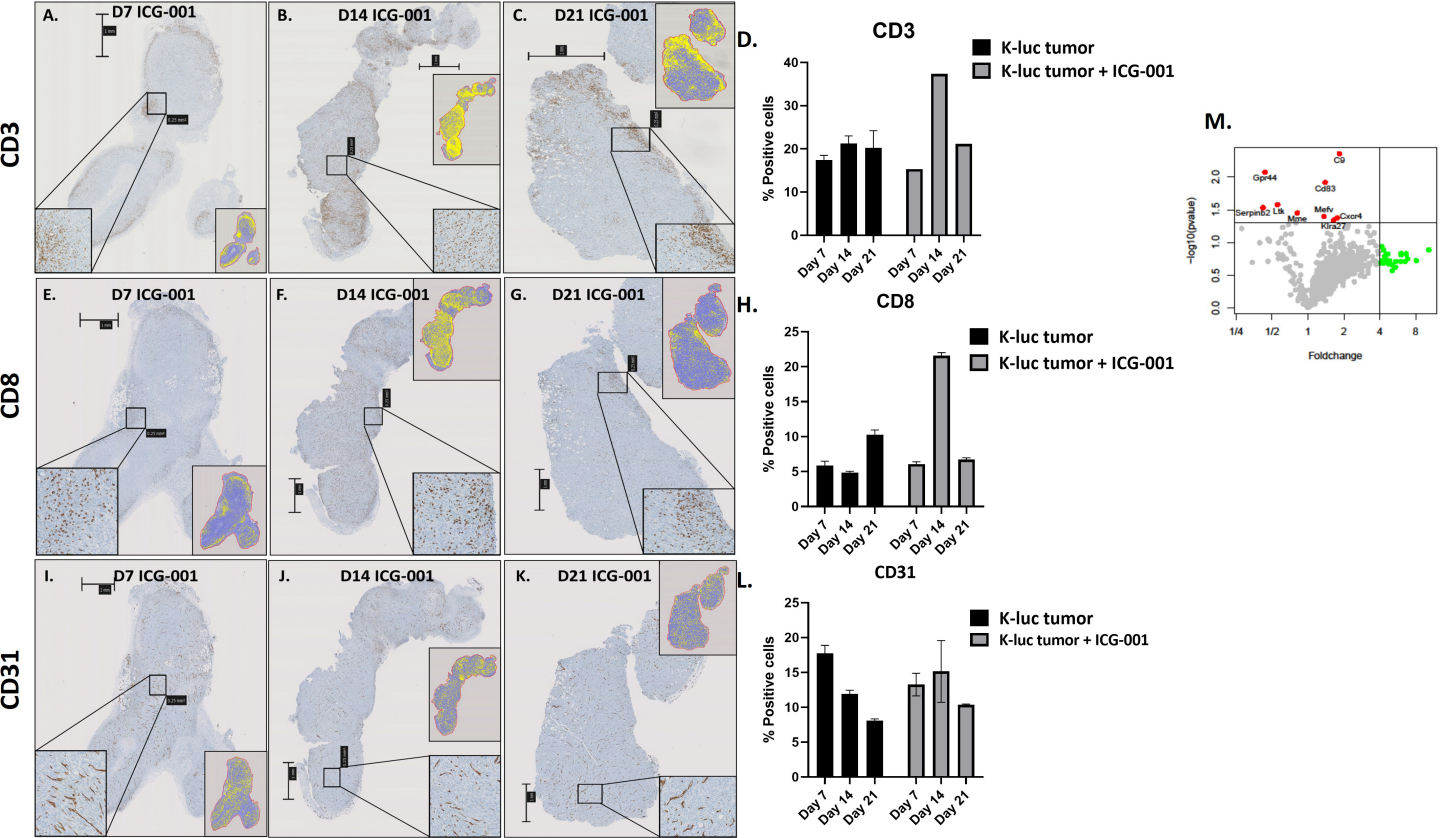
IHC analysis of immune cells in syngeneic models of subcutaneous glioma (K-Luc). **(A–C, E–G, I–K)** Mice bearing subcutaneous K-luc glioma tumors were treated with ICG-001 (50 mg/kg/day) for 7, 14, or 21 days using Alzet minipumps. At the end of treatment, tumor tissue was excised and IHC stained for CD3 cells to evaluate recruitment and patterns of CD3, CD8, and CD31 distribution. **(D, H, L)** Quantification of tumor coverage was performed using QuPath for mouse CD3, CD8, and CD31 positive cells on days 7, 14, 21 post minipump implantation. Pairwise comparison was performed to access the CD31 expression on days 7, 14 and 21 (P-value 0.314, 0.624, 0.0016 respectively). Control animals were treated with no pumps. Scale bars are SD of duplicate sections (n=4). **(M)** NanoString analysis of mouse immune panel genes in subcutaneous K-Luc tumors at days 7 and 21, as compared with untreated controls (untreated tumors were also collected on days 7 and 21).

We anticipated that modulation of the cold TME by ICG-001 should enhance the recruitment of T cells into the TME, because upregulation of the Wnt pathway has been shown to be involved in immune evasion (**Figure 5**) (49). To test this hypothesis, we used a subcutaneous model of syngeneic K-Luc glioma cells implanted into C57BL/6 mice. Mice received 1×10^6^ tumor cells and were either untreated or treated with ICG-001 on day 7 after tumor implantation (delivered by subcutaneous Alzet minipumps, 50 mg/kg/day) (**Figures 5A–C**). The tumors were harvested and evaluated for CD3, CD8, and CD31 by immunohistochemistry (IHC) on days 7, 14, and 21 post-Alzet minipump implantation (ICG-001 release by Alzet pump continues for 28 days post implantation) and compared to the tumors from untreated controls that were harvested on the same days (**Figures 5E–G, I–K**). We observed an increase in host immune cells, including CD3^+^ and CD8^+^ T cells, infiltrating tumors by day 14 post-ICG-001 treatment (**Figures 5D, H**). A time-dependent increase in CD31 expression was also observed, when compared with controls (**Figure 5L**).

NanoString gene expression analysis of subcutaneous tumors from ICG-001-treated mice (daily release of ICG-001 by Alzet pump is 50 mg/kg/day) versus untreated (harvested on the same days 7, 14, 21) tumors identified genes that were significantly upregulated (p ≤ 0.05), such as C9, CD83, Gpr44, Cxcr4, and others (**Figure 5M** and **Supplementary Table 1**) with a demonstrated fold-change increase (>=4) such as Dmbt1, Nsr1, Irf4, Klrg1, Gzmb, and others (**Figure 5M**). On day 7 and 14 post Azlet pump implantation, we observed upregulation of tumor suppressor gene DMBT1 and the chemotactic factor CCL24, which displays chemotactic activity on resting T lymphocytes, and both GZMA and GMZB, which are associated with cytotoxic T cell activation on day 7 and 14 post Alzet pump implantation (**Supplementary Table 1**) was observed. These results further support our hypothesis that specific downregulation of Wnt/CBP/β-catenin signaling along with reprogramming of the TME may enhance the efficacy of tumor immunotherapy, increase recruitment of host T-cells, and improve immunotherapy to solid tumors (50).

### 3.6 ICG-001 treatment regimen added before or after CAR T cell therapy in intracranial, syngeneic K-luc glioma

**Figure 6:**
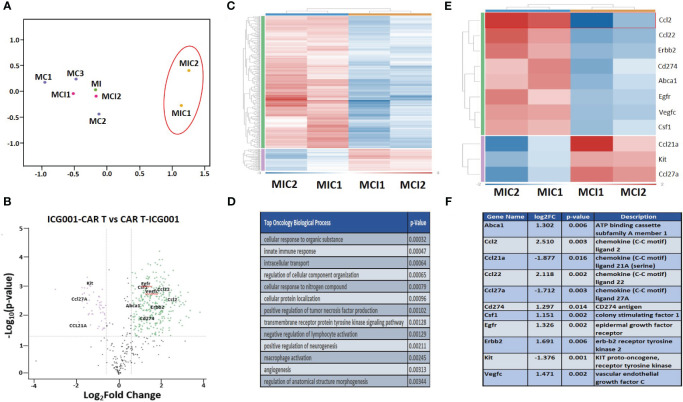
NanoString analysis of nCounter mouse Pan Cancer immune profile gene expression within ICG-001, CAR T, CAR T + ICG-001 and ICG-001 + CAR T treatment groups. **(A)** Multidimensional scaling (MDS) plot for the samples treatments with ICG001[MI] (ICG001 only), 3 treatments with CAR Ts [MC] (CART only), 2 treatments with ICG001 and then CAR Ts [MIC] (ICG001 first then CART), and 2 treatments with CAR Ts and ICG001 [MCI] (CART first then ICG001). **(B)** Volcano plot showing difference in expression of |Fold Change|>1.5 & p-value<0.05 between ICG001-CAR T and CAR T-ICG001 treated mice, with the x-axis showing log2 fold change using cutoff and the y-axis showing the negative log10 of p-value for the 11 selected mouse brain tumor genes of interest. **(C)** Heatmap of differential expression gene comparison between ICG001 + CAR T and CAR T + ICG001 using filtering |FC |≥ 1.5 & p-value ≤ 0.05 label highlighted. **(D)** The top statistic significant ROSALIND oncology collection biologic process based on the differential expression gene expression comparison between ICG001 + CAR T and CAR T + ICG001. **(E)** The heatmap of differential expression gene comparison between ICGCART & CARTICG for the 11 selected brain tumor genes of interest which are also labelled on Volcano plot. **(F)** The description and log2(fold change) & p-value for the 11 selected brain tumor genes of interest.

To understand the effect of ICG-001 on tumor metabolism alone and in combination with IL13Rα2–CAR T cells, we established orthotopic immunocompetent mouse models of syngeneic glioma using K-Luc glioma cells and engineered murine IL13Rα2-targeted CAR T cells (51). The murine IL13Rα2- targeted CAR T cells (mIL13BBζ CAR T cells) were characterized by FACS and contained comparable numbers of CD4+ and CD8+ T-cell subsets, with a mixture of early memory (CD62L+) and effector (CD62L−) T-cell populations, as described previously (51). We developed intracranial tumors by administration of K-luc glioma cells (0.1×10^6^) into the right frontal lobe of C57BL/6 mice. The K-luc tumor line derived from a spontaneous glioma arising from *Nf1, Trp53* mutant mice (KR158) is poorly immunogenic, as indicated by its unresponsiveness to anti–PD-1 checkpoint therapy (52). This line has been further engineered to express the murine IL13Rα2 and used to recapitulate invasive glioma in syngeneic mouse models. On day 14 post-tumor implantation, mice received intratumoral administration of CAR T cells at a sub-therapeutic dose (1×10^6^) (cF11240 Mouse T cell mIL13-mCD8h-mCD8tm3-m41BB-mZeta-T2A-mCD19t(CO)_MSCV) (n=14). Treatment groups were as follows: 1) Tumor only [GR1]; 2) Tumor + ICG-001 (D16-[MI-GR2]); 3) Tumor + CAR Ts (D14–[MC-GR3]); 4) Tumor + CAR Ts (D14) and ICG-001 (D16—[MCI-GR4]) (n=14); 5) Tumor + ICG-001 (D7)+ CAR Ts (D14–[MIC-GR5]). Tumor-bearing mice in GR5 were pretreated with ICG-001 (starting on Day 7 post tumor implantation for 28 days) 1 week prior to CAR T administration [MIC-GR5]. ICG-001 was given via Alzet subcutaneous minipump delivering a dose of 50 mg/kg/day over 28 days. Otherwise, pumps were implanted on day 16, 2 days post CAR T administration (D14) in the treatment group GR4. Mice were monitored until they developed tumors larger than 0.5 cm^2^. Normalized RNA concentration per tumor tissue (1 mg) was used for NanoString analysis. NanoString pathway analysis revealed the top genes that were up- or downregulated in CAR T vs CAR T + ICG-001 groups using a mouse immune panel to detect metabolic changes in mouse K-luc tumors and to visualize endogenous immune cells infiltration upon inhibition of Wnt with ICG-001 (nCounter_Mouse_PanCancer_Immune_Profiling_Panel). Once tumors reached the predetermined criteria for euthanasia, they were harvested, isolated, and subjected to IHC and NanoString analysis to detect differentially expressed genes in ICG + CAR T[MIC-GR5] versus CAR T + ICG-001[MCI-GR4] (**Figure 6**). Rosalind analysis (https://www.rosalind.bio) revealed that gene expression of all CAR T, ICG001, and CAR T+ ICG-001 treated tumors were clustered together on the MDS plot, whereas ICG-001 pretreated and CAR T treated tumors demonstrated very different gene expression profiles on the MDS plot (**Figure 6A**). The volcano plot shows difference in expression of selected genes between ICG-001 + CART versus CART+ ICG-001 with the x-axis of log2 fold change and y-axis of the negative log10 of p-value. We observed downregulation of Kit, CD27A, and CCl23A, and upregulation of CCL2, VEGFC, Erbb2, Cd274, CCL22, and CSF1, contributing to improved T cell trafficking (**Figure 6B**). The full list of differentially regulated genes is presented in **Supplementary Table 1**. The top up and downregulated genes are displayed in **Figures 6E, F** and the top biological processes affected are displayed in **Figure 6D**. These changes demonstrate the reprogramming of tumors pre-treated with ICG-001 and then CAR T and changes in metabolic pathways and genes, shifting to a more differentiated, immune responsive tumor.

### 3.7 ICG-001 treatment regimen affects immune cell infiltration when added before or after CAR T cell therapy: cell line profiling using NanoString analysis

**Figure 7:**
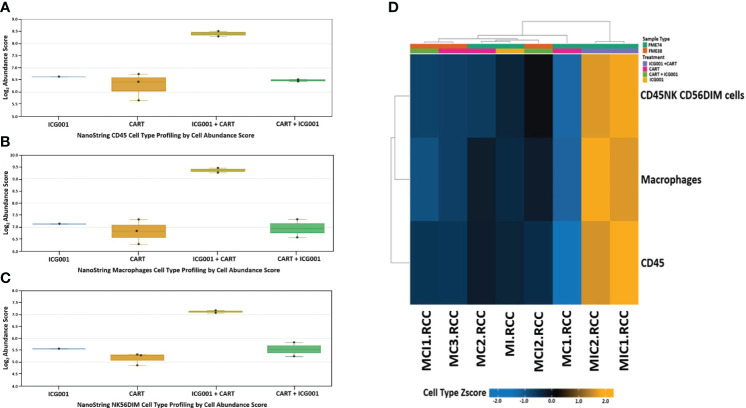
NanoString nCounter mouse cell line PanCancer immune profiling: Genes previously shown to be characteristic of various cell populations by measuring cells population abundance based on the NanoString Cell Type Profiling Module. **(A–C)** Bar graph charts showing abundance scores of CD45, NK-CD56, and Macrophage cell type populations across all samples for genes that are characteristic and grouped by cell population abundance. **(D)** Heat map of gene clustering and cell profile in groups treated with ICG001 (MI), CAR T + ICG001 (MCI1, MCI2), ICG001+ CAR Ts (MIC1, MIC2), and CAR Ts only (MC1, MC2, MC3). Genes previously shown to be characteristic of CD45, NK-CD56, and Macrophage cell population abundance across all samples for ICG001+ CAR Ts (MIC1, MIC2) shows positive Z-score differently comparing from samples of ICG001 (MI), CAR T + ICG001 (MCI1, MCI2), and CAR Ts only (MC1, MC2, MC3) with negative Z-score.

Next, we compared CD45, Macrophages, and NK cell infiltration within tumors treated with 2 different schedules of administration of ICG-001 [CAR T + ICG001[GR4] versus ICG-001 + CAR Ts[GR5]]. We compared groups treated with ICG-001 only, CAR T only, and CAR T [D14] + ICG-001[D16] in comparison with ICG-001[D7] only or CAR T only treated groups[D14]. We found increased infiltration of CD45+ macrophages and NK-CD56dim (natural killer cells responsible for cytolytic activity and target cell killing) cells in the ICG-001+CAR T group [GR5] (**Figures 7A–D**). Cell type profiler analysis identified clearly different immune cell infiltration in the GR5 [ICG-001 + CAR T] treated group when compared with controls (GR1[ICG001], GR2 [CAR T], and GR4 [CAR T + ICG001] treated groups). We also observed an increase in the expression of the gene PTPRC encoding CD45RA that plays role in lymphocyte function, and an increase in KLRG1- cells contributing to an increase in all memory T cell lineages, including peripheral memory and tissue-resident memory cells, which should enhance long-term protective immunity (53) (**Supplementary Figure 4A**). Upregulation of the SELL (aka E-selectin CD62L) gene T_scm_ CD45RA^+^, IL7R^+^, CCR7^+^, CD62L^+^, KLRG^-^ was found within a group treated with ICG-001 first and then CAR T [ICG001 + CAR T], which is a marker associated with the T cell memory phenotype (**Supplementary Figure 4B**). We also found upregulation of IL7R- Interleukin-7 receptor (IL7R), which provides the potential for long-term survival of both CD62L^high^ central memory T cells and Th1 effector cells (**Supplementary Figure 4C**) (54). CCR7 is important in the migration of memory CD8+ T cells and survival in their niches (**Supplementary Figures 4D, E**).

### 3.8 Combination of ICG-001 with CAR T cells enhances cytotoxicity to glioma *in vitro*

We next tested the efficacy of a titrated dose of CAR T cells ± ICG-001 to induce tumor cell killing *in vitro* (**Supplementary Figure 5**). We evaluated the combination of HER2-engineered CAR T (HER2-CAR T) cells and PBT106 tumor cells (high expressing HER2) in combination with IGC-001 using an *in vitro* co-culture assay. ICG-001, in combination with HER2-CAR T cells, demonstrated synergistic cytotoxicity in several patient-derived glioma lines that expressed high levels of the tumor antigen HER2 (**Supplementary Figure 5**). ICG-001 combined with HER2-CAR T cells demonstrated synergistic killing in a CAR T cell concentration-dependent fashion (**Supplementary Figure 5A**). Enhanced killing was observed with the combination of HER2-CAR T cells and ICG-001 at effector/target cell ratios of both 1:2 and 1:4 (**Supplementary Figures 5B, C**). Furthermore, CBP/β-catenin antagonism with ICG-001 was not toxic to T cells *in vitro* or *in vivo*, providing important insights for the advancement of CAR T cell therapy combined with CBP/β-catenin antagonism for the treatment for brain tumors (**Supplementary Figure 6**).

## 4 Discussion

Tumors evolve a variety of mechanisms to escape immune surveillance, including evading immune recognition, promoting regulatory cell expansion, coopting inhibitory signals, and secreting suppressive factors. Impressive results in a subset of cancers have been obtained using immunotherapies such as checkpoint inhibitors (i.e. anti-CTLA4, anti-PD-1, and anti-PD-L1) and CAR T therapies. Still, only a subset of patients respond to these immunotherapies, and the response rate in certain tumor types, including glioma, is very low, and relapse remains a significant concern (55). The Wnt/β-catenin signaling pathway, which is a critical regulator of both somatic stem cells and cancer stem/tumor initiating cells, has been correlated with resistance to radiation and cytotoxic and targeted chemotherapy. Wnt/β-catenin pathway signaling is also correlated with immunotherapy resistance in melanoma (15, 56, 57) and across other tumor types (12). Tumor-intrinsic Wnt/β-catenin signaling mediates cancer immune evasion by preventing T-cell and/or dendritic cell infiltration, migration, and function, and thereby resistance to immunotherapies (12, 13, 16, 58).

Wnt/β-catenin signaling also plays an essential role in the development and maintenance of multiple organ systems, including the brain. A dichotomous role for Wnt signaling in stem cells, organ development, maintenance, and repair is a commonly observed phenomenon, with Wnt signaling playing critical roles in the processes of both cell proliferation and differentiation. We have previously demonstrated that aberrant Kat3 coactivator usage (i.e., enhanced CBP usage at the expense of p300 by β-catenin) is responsible for the improper termination of the wound healing process by maintaining epithelial cell proliferation and inhibiting differentiation (59). Small molecule CBP/β-catenin antagonists, which target a fundamental control switch in stem cell biology, can overcome cytotoxic or targeted chemotherapy and immunotherapy resistance (28, 60) through forced symmetric differentiation, thereby eliminating CSCs while having beneficial effects on the normal somatic stem cell population, which preferentially divides asymmetrically (25, 59). By binding with high affinity (K_d_~1nM) and specificity to the N-terminus of CBP, the small molecule CBP/β-catenin antagonist ICG-001 can safely correct aberrant Wnt signaling to initiate differentiation via p300/β-catenin transcription (25).

The concept of combining a specific small molecule CBP/β-catenin antagonist with immunotherapy to reverse Wnt/β-catenin-mediated cancer immune evasion has been previously explored preclinically in NAFLD-associated liver cancer (60) and colorectal cancer to liver metastasis (28). However, using a CBP/β-catenin antagonist to induce the differentiation of GSC without damaging effects on somatic stem cells (41) is novel. Although ICG-001 has high biochemical specificity for the N-terminus of CBP, its mechanism of action is highly pleiotropic via the modulation of enhancers and super-enhancers in multiple cell types beyond tumor cells themselves, including mesenchymal, endothelial, and immune cell populations (40, 59, 61).

In using human glioma xenograft models with patient-derived tumors in [NSG NOD-*scid* IL2Rgamma^null^] mice, we previously found that IL13BBζ-CAR T cells improved anti-tumor activity and T cell persistence as compared to first-generation IL13ζ-CAR CD8(+) T cells that had shown evidence for bioactivity in patients (21). However, our studies were not limited by the tumor target-specific CARs, and can be expanded to various CAR therapies as well (62). To overcome known limitations of the use of CAR T cell therapy for glioma, such as antigen escape and the need for increased T cell persistence and potency, in the current manuscript, we tested a novel combination therapeutic approach with the Wnt/CBP/β-catenin antagonist ICG-001 to enhance the expression of genes involved in antigen presentation and the adaptive immune response.

Establishing the proof-of-concept that ICG-001 can safely target aberrant Wnt/β-catenin signaling in glioma in combination with CAR T cell therapy could provide a novel means of broadening the tumor response to CAR T cell therapy while decreasing the resistance and relapse arising post-CAR T cell therapy in solid tumors. However to date, targeting aberrant Wnt signaling clinically with anything other than a specific CBP/β-catenin antagonist has demonstrated significant on-target associated toxicities (59). We also separately analyzed the immune subsets and Wnt/β-catenin pathway activation in the TME of brain tumors [BTs] in preclinical models using novel techniques such as gene expression profiles and proteomics to elucidate downstream target genes.

Key genes involved in glioma cell differentiation, including ASCL1 and PTPRZ1-MET, have been shown to contribute to the development of a glioma stem cell phenotype, which is thought to be the source of resistance and relapse after initial treatment with checkpoint inhibitors (63, 64). Furthermore, reprogramming the immune landscape with a single gene in non-cancer cells identified S100A8 as a regulator of an immune suppressive T and myeloid cell subtype (65). We have also demonstrated infiltration of CD45, Macrophages, and NK cells into the tumor stroma, and upregulation of KLRG1, Sell, Ccr7, and IL7r upon the treatment with ICG-001 and then CAR Ts (66).

## 5 Conclusion

These studies have demonstrated that CBP/β-catenin antagonists induce significant differences in the expression of genes and proteins involved in proliferation and differentiation of tumor cells and of critical metabolic pathways. These promising studies provide the basis for future development of this multi-targeted approach, with the goal of developing a breakthrough treatment for patients with brain tumors.

## Data availability statement

The original contributions presented in the study are publicly available. This data can be found via the following link: https://www.ncbi.nlm.nih.gov/geo/query/acc.cgi?acc=GSE252155.

## Ethics statement

The animal study was approved by Institutional AnimalAnimal Care and Use Committee 18059. The study was conducted in accordance with the local legislation and institutional requirements.

## Author contributions

MG: Conceptualization, Data curation, Resources, Supervision, Writing – original draft, Writing – review & editing, Investigation, Methodology, Project administration. JH: Data curation, Formal analysis, Investigation, Methodology, Writing – original draft, Validation, Visualization. EMa: Investigation, Methodology, Validation, Visualization, Writing – review & editing. HN: Data curation, Investigation, Methodology, Writing – review & editing. VA: Data curation, Formal analysis, Investigation, Methodology, Writing – review & editing. NC: Data curation, Formal analysis, Writing – review & editing. RQ: Data curation, Methodology, Writing – review & editing. CN: Data curation, Methodology, Writing – review & editing. EMe: Data curation, Investigation, Methodology, Writing – review & editing. BA: Data curation, Funding acquisition, Investigation, Methodology, Writing – review & editing. RS: Data curation, Methodology, Project administration, Writing – review & editing. MO: Data curation, Writing – review & editing, Formal analysis. HY: Methodology, Writing – review & editing, Software, Data curation, Funding acquisition. RR: Formal analysis, Investigation, Writing – review & editing, Conceptualization, Methodology, Software, Validation. NB: Investigation, Formal analysis, Writing – review & editing. YCY: Data curation, Formal analysis, Investigation, Methodology, Software, Visualization, Writing – review & editing. CB: Conceptualization, Data curation, Investigation, Supervision, Writing – review & editing, Formal analysis, Funding acquisition, Resources. MK: Data curation, Formal analysis, Writing – review & editing, Conceptualization, Investigation, Project administration, Supervision, Validation, Visualization, Writing – original draft.
