# Supplementary material for: Targeting Wnt signaling for improved glioma immunotherapy
Source: Front Immunol. 2024 Feb 21;15:1342625. doi: 10.3389/fimmu.2024.1342625 (PMC10915090; doi:10.3389/fimmu.2024.1342625)
Supplement: Supplementary file 1 [file Table_1.docx]

| **Genes with est pvalue <=0.05. (red)** | | | | **Genes with est foldchange >=4. (green)** | | |
| --- | --- | --- | --- | --- | --- | --- |
| **Gene** | **Foldchange** | **p-value** |  | **Gene** | **Foldchange** | **p-value** |
| **C9** | **1.83** | **0.0044** |  | **Dmbt1             1** | **10.33** | **0.13** |
| **Cxcr4** | **1.76** | **0.042** |  | **Ncr1** | **8.09** | **0.19** |
| **Klra27** | **1.64** | **0.046** |  | **Irf4** | **6.86** | **0.18** |
| **Cd83** | **1.40** | **0.012** |  | **Klrg1** | **6.67** | **0.15** |
| **Mefv** | **1.36** | **0.040** |  | **Lyve1** | **6.53** | **0.20** |
| **Mme               0** | **0.81** | **0.035** |  | **Cd7** | **6.05** | **0.16** |
| **Ltk** | **0.56** | **0.026** |  | **Gzmb** | **6.04** | **0.15** |
| **Gpr44** | **0.44** | **0.0084** |  | **Card11            6** | **6.02** | **0.20** |
| **Serpinb2          0** | **0.42** | **0.029** |  | **Cd6** | **5.47** | **0.20** |
|  |  |  |  | **Cmah** | **5.40** | **0.24** |
|  |  |  |  | **Prf1** | **5.11** | **0.22** |
|  |  |  |  | **Cfd** | **5.09** | **0.27** |
|  |  |  |  | **Lck** | **4.84** | **0.19** |
|  |  |  |  | **Pou2f2            4** | **4.76** | **0.22** |
|  |  |  |  | **Ccr3** | **4.70** | **0.15** |
|  |  |  |  | **Ebi3** | **4.53** | **0.15** |
|  |  |  |  | **Klrc2** | **4.45** | **0.18** |
|  |  |  |  | **Cd2** | **4.43** | **0.20** |
|  |  |  |  | **Itk** | **4.41** | **0.16** |
|  |  |  |  | **Pycard** | **4.33** | **0.21** |
|  |  |  |  | **Il13** | **4.30** | **0.13** |
|  |  |  |  | **Txk** | **4.24** | **0.20** |
|  |  |  |  | **Flt3l** | **4.21** | **0.19** |
|  |  |  |  | **Fasl** | **4.16** | **0.12** |
|  |  |  |  | **Ltb** | **4.07** | **0.21** |
