# Supplementary material for: Targeting Wnt signaling for improved glioma immunotherapy
Source: Front Immunol. 2024 Feb 21;15:1342625. doi: 10.3389/fimmu.2024.1342625 (PMC10915090; doi:10.3389/fimmu.2024.1342625)
Supplement: Supplementary file 4 [file DataSheet_1.pdf]

Supplementary Material

Targeting WNT Signaling for Improved Glioma Immunotherapy

- *M Gutova[1], JC Hibbard[2], Eric Ma[2], Natri M. Heini[3], Vikram Adhikarla[4], Nyam-Osor Chimg[5], Runxiang Qiu[1], Cu Nguye[5], Elizabeth Melendez[5], B Aguilar[2], R Starr[2], Holly Yin[5], Russell Rockne[4], Masaya Ono[6], Nicholas E. Banovich[3], Yate-Ching Yuan[4], Christine E. Brown[2] and Michael Kahn[5]*

*** Correspondence:**

Margarita Gutova, MD

Associate Research Professor,

Department of Stem Cell Biology and Regenerative Medicine

Beckman Research Institute, City of Hope

1500 East Duarte Road

Duarte CA 91010

E-mail: MGutova@coh.org

Supplemental Figure 1.

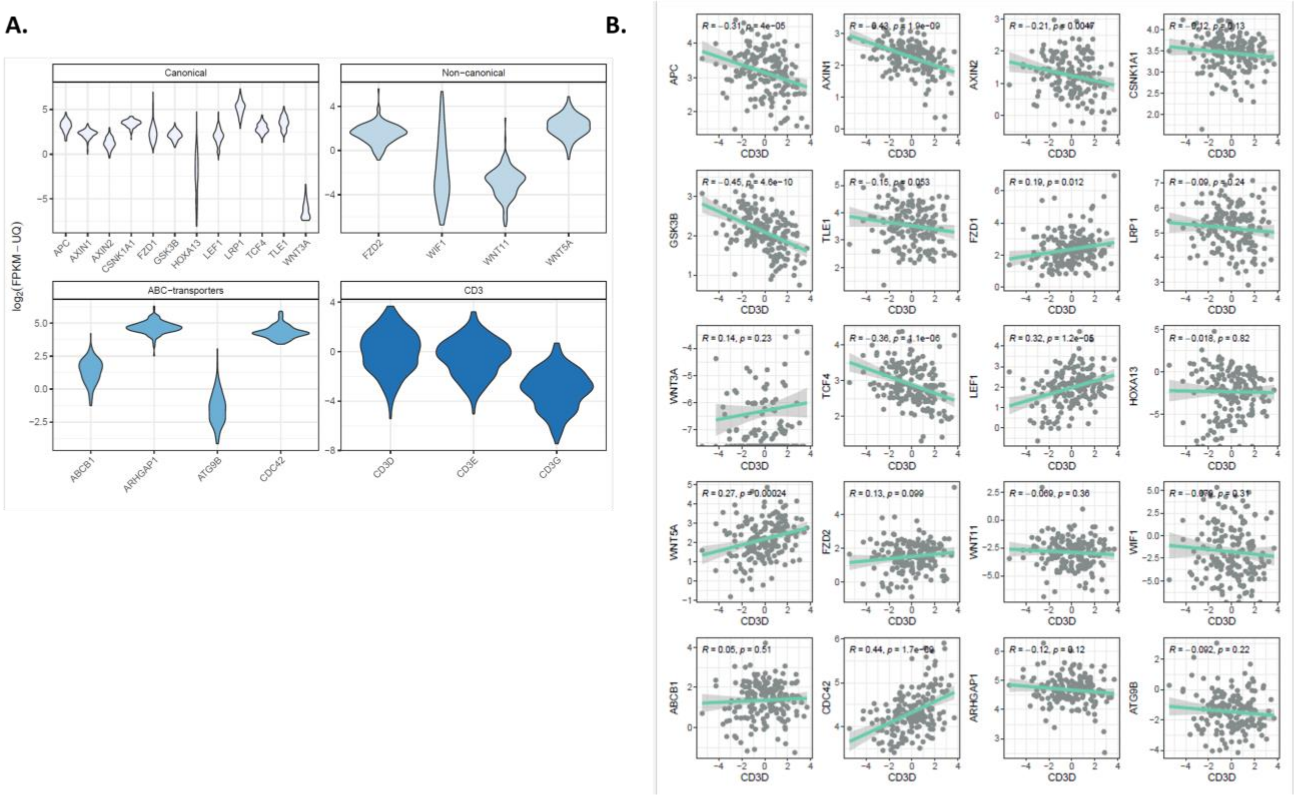

Figure S1. TCGA (the Cancer Genome Atlas) analysis of WNT catenin pathway genes (**A**) in glioma and correlation with CD3 cell infiltration (**B**). Gene expression levels were visualized using Fragments Per Kilobase of transcript per Million mapped reads Upper Quartile (FPKM-UQ) values.

Supplemental Figure 2.

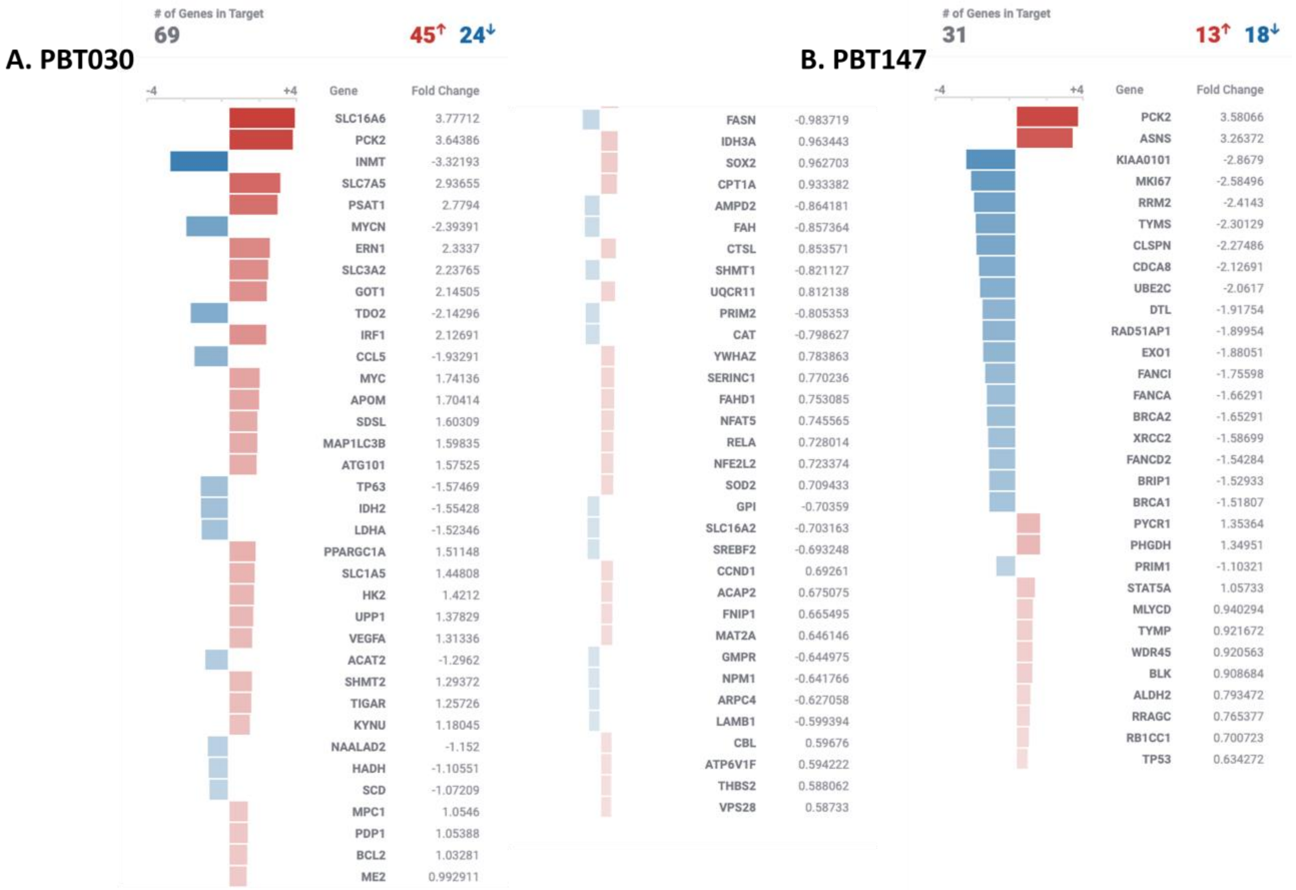

Figure S2. (**A, B**) Genes regulated by ICG-001 in glioma cell lines PBT030 (**A**) and PBT147(**B**). A full list of up and downregulated genes with > 1.5, > -1.5 and p-Adj 0.05 filter is shown.

Supplemental Figure 3.

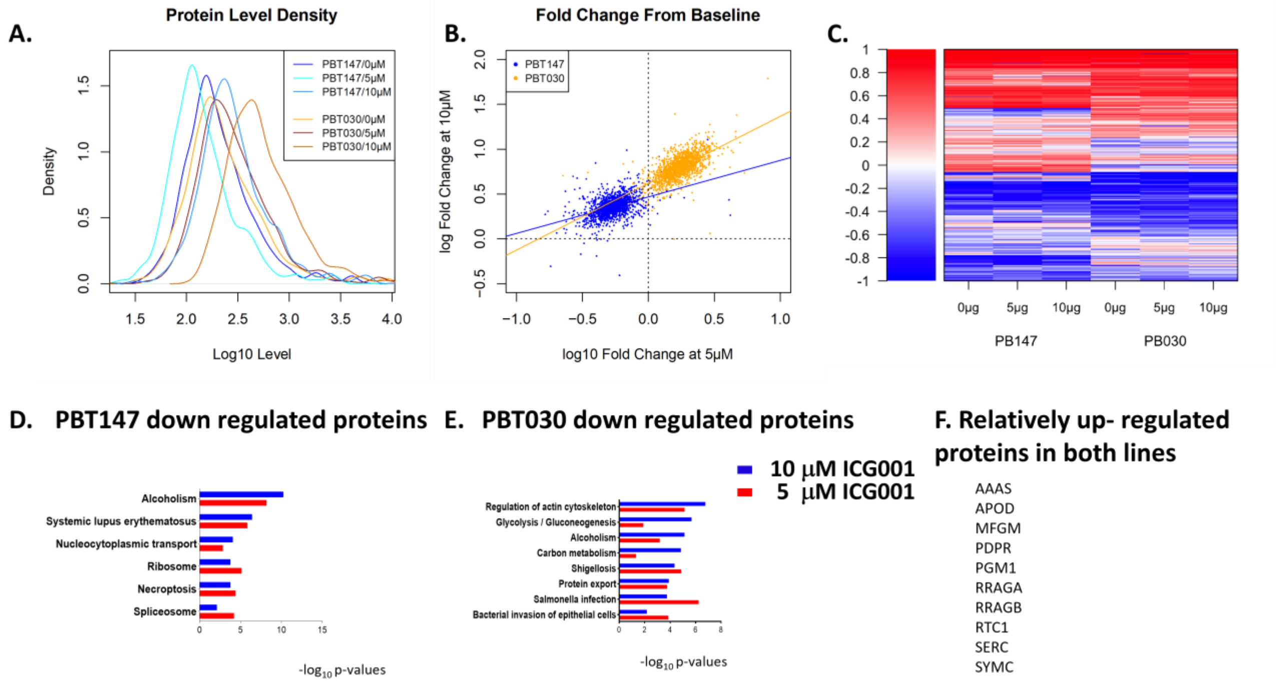

Figure S3. Protein expression analysis of PBT147 and PBT030 cell lines treated with ICG-001 at 0, 5, and 10 µM. (**A**) Protein density Plot. (**B**) Fold change from baseline. (**C**) Heat map. (**D, E**) downregulated proteins upon treatment with ICG-001.

Supplemental Figure 4.

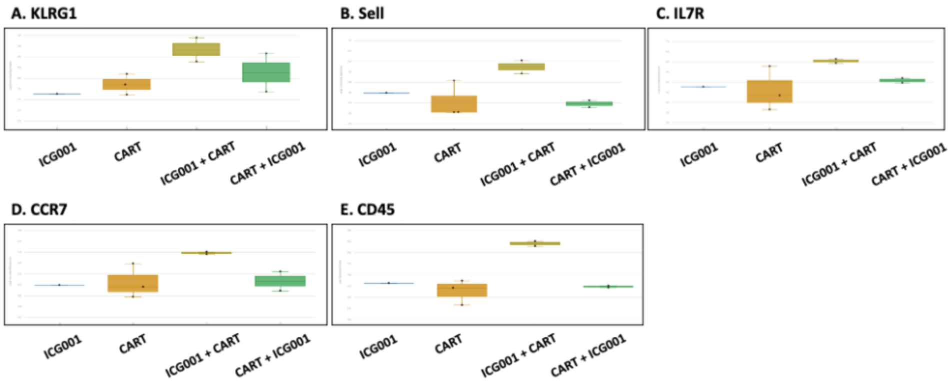

Figure S4. Cell type analysis in K-Luc tumors treated with ICG-001 and CAR Ts.

Supplemental Figure 5.

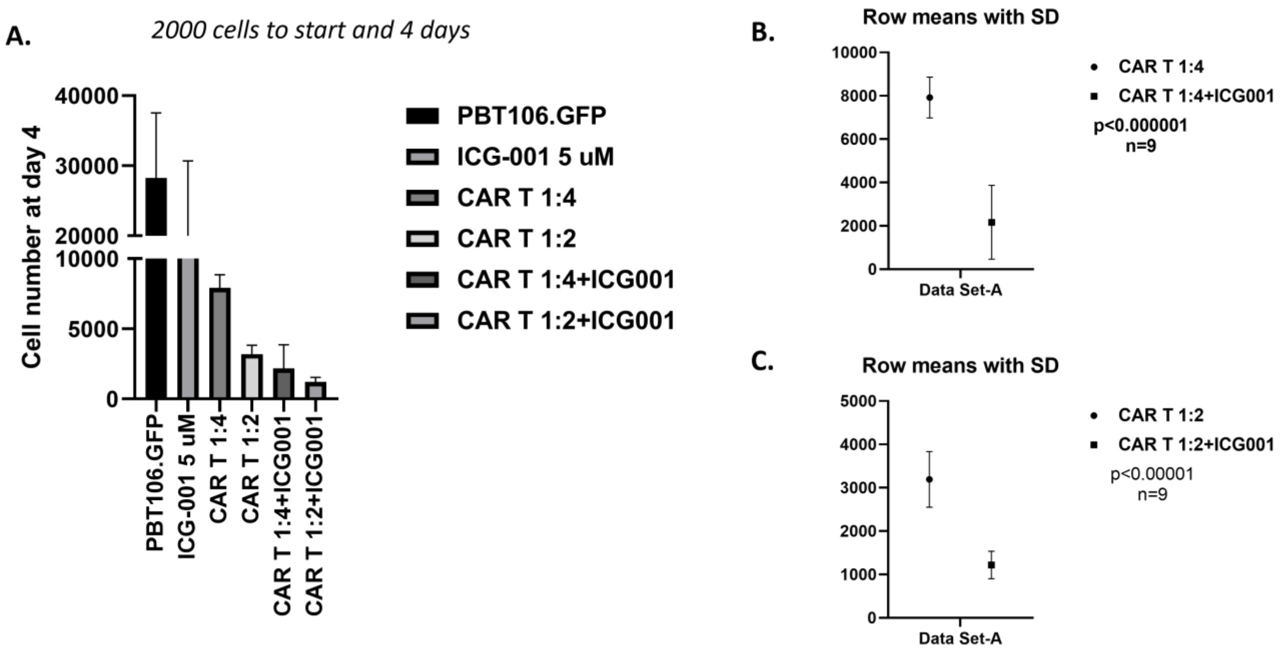

Figure S5. Growth kinetics of PBT017 (2,500 cells/well) treated with or without HER2-CAR T cells (500 cells/well) and with or without ICG-001 (0-5 µM). Experiment performed in quadruplicate, and error bars indicate SD.

Supplemental Figure 6.

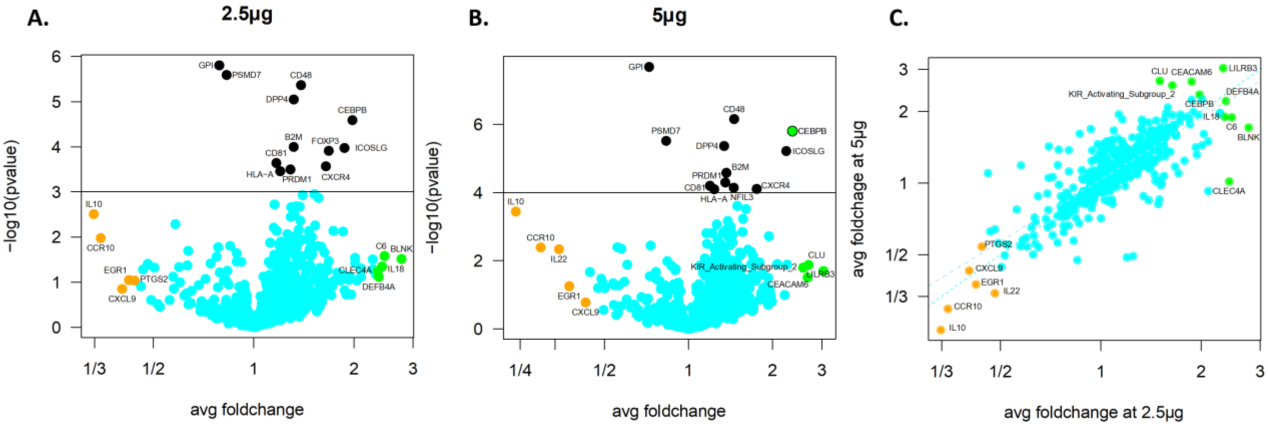

Figure S6. T cells treated with ICG001 2.5 and 5 μM of ICG001 for 24 or 72 h.

Supplemental Figure 7.

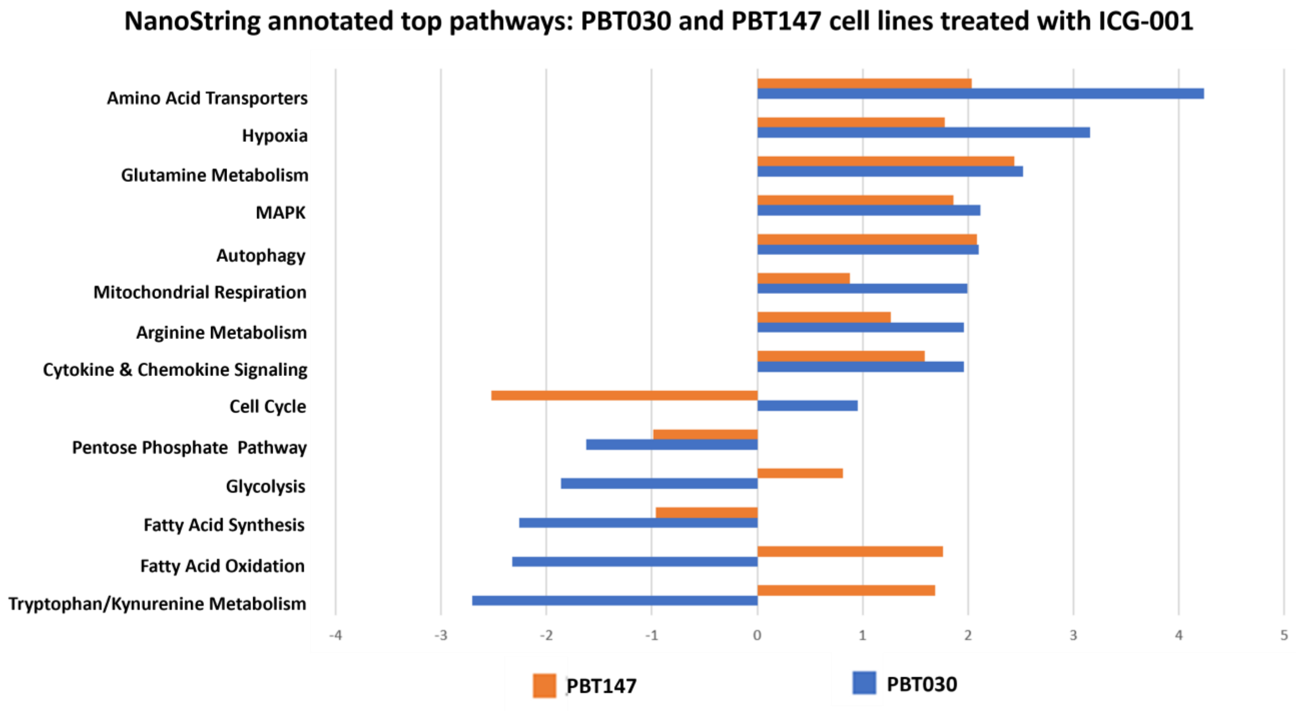

Figure S7. Top pathways annotated in PBT030 and PBT147 cell lines treated with ICG001.

Supplemental Figure 8.

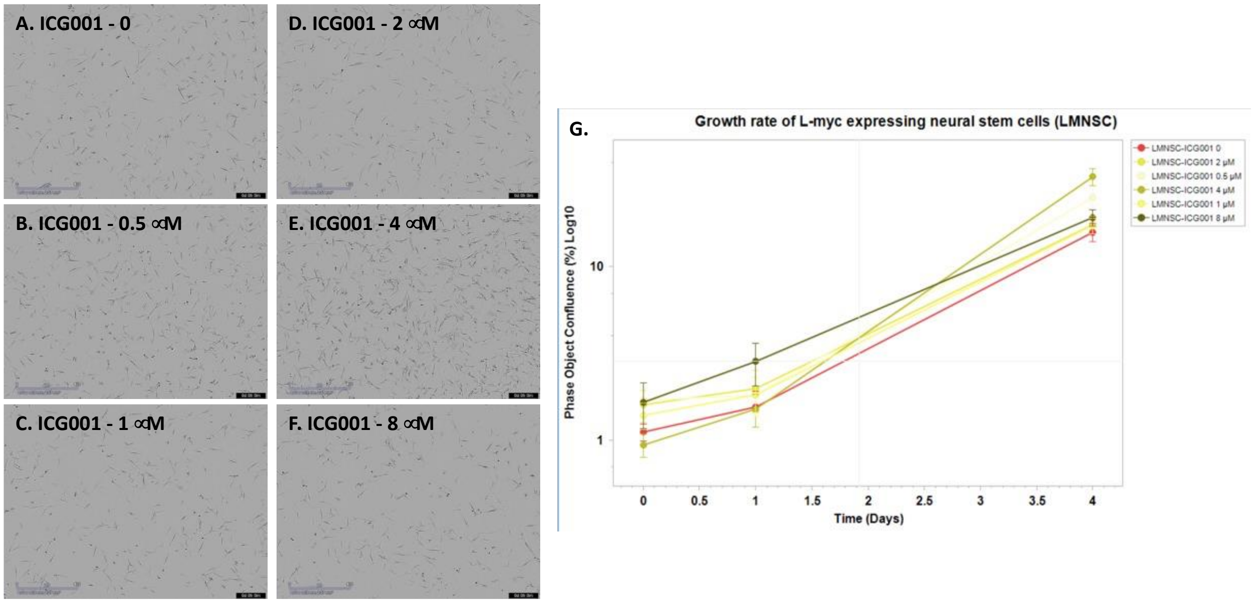

Figure S8. Treatment of normal neural stem cells immortalized with *L-MYC* gene (LMNSC008) with ICG001 did not result in any toxicity for over 4 days (dose of ICG001 was 0, 4, 8 μM), as demonstrated by IncuCyte images (**A-F**) and representative growth curve (**G**).

Supplemental Figure 9.

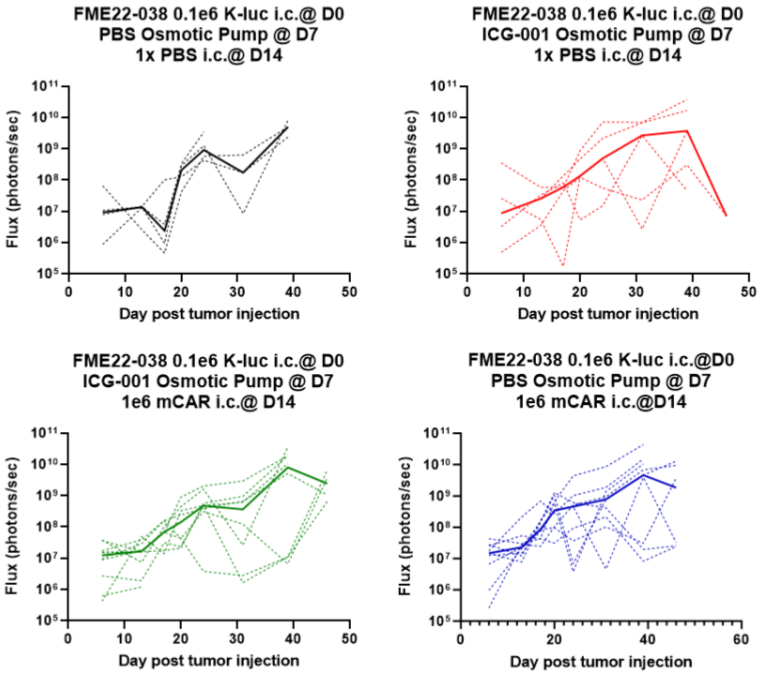

Figure S9. BLI (bioluminescence imaging) images of intracranial K-Luc tumors, treated with ICG001 pump, starting on day 7 and followed by CAR T therapy on day 14.

Supplemental Figure 10.

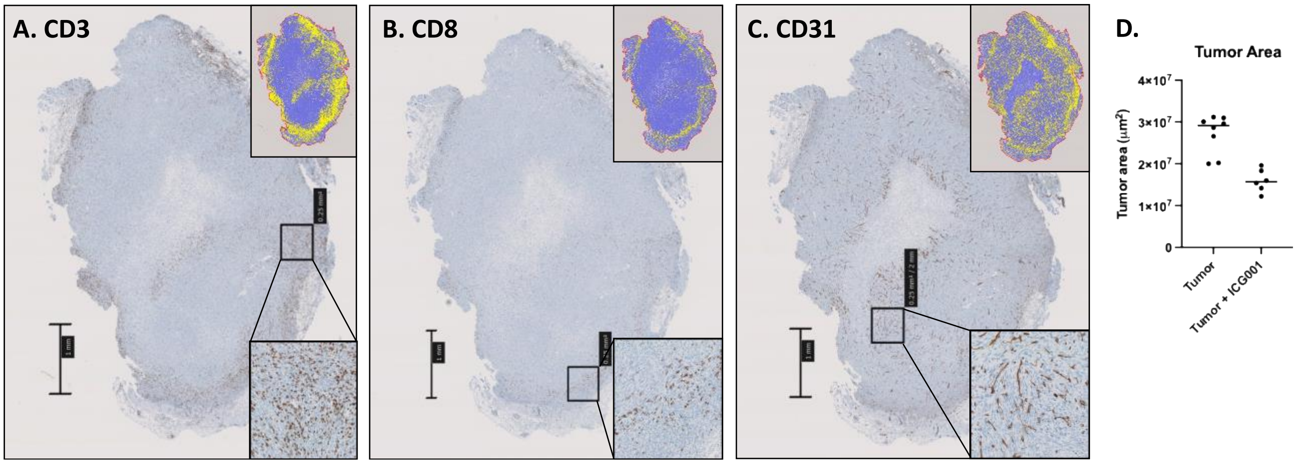

Figure S10. IHC images of K-Luc subcutaneous tumors. (A-C) Images represent no ICG001 treated subcutaneous tumors. IHC was performed for mouse CD3 (A), mouse CD8 (B) and mouse CD31 (C). Total tumor area was calculated using QuPath of H&E sections (D).
